# Effect of Dietary Aryl Hydrocarbon Receptor Ligands on Indoxyl Sulfate-Induced Endothelial Activation

**DOI:** 10.3390/toxins18070298

**Published:** 2026-07-10

**Authors:** Flora Lefevre, Rania Chermiti, Julien Cebile, Nathalie McKay, Stanislas Bataille, Stéphane Burtey, Laetitia Dou

**Affiliations:** 1 C2VN, Aix-Marseille University, INSERM, INRAE, 13385 Marseille, France; raniachermiti123@gmail.com (R.C.); julien.cebile@etu.univ-amu.fr (J.C.); nathalie.mc-kay.1@univ-amu.fr (N.M.); stephane.burtey@univ-amu.fr (S.B.); laetitia.dou@univ-amu.fr (L.D.); 2 Centre de Néphrologie et Transplantation Rénale, Assistance Publique des Hôpitaux de Marseille, Hôpital Conception, 13005 Marseille, France; 3 Phocean Nephrology Institute, Clinique Bouchard, ELSAN, 13005 Marseille, France; stanislas.bataille@gmail.com

**Keywords:** aryl hydrocarbon receptor, chronic kidney disease, indolic uremic toxins, dietary AhR ligands

## Abstract

Patients with chronic kidney disease (CKD) are exposed to high levels of uremic toxins and have an increased risk of cardiovascular disease. Among these toxins, indolic compounds such as indoxyl sulfate (IS) are predictors of cardiovascular events and mortality in CKD patients and induce a procoagulant and proinflammatory vascular phenotype through activation of the aryl hydrocarbon receptor (AhR). Targeting AhR activation by indolic toxins may therefore help prevent cardiovascular complications in CKD. To this end, we investigated in vitro whether natural dietary AhR ligands (galangin, quercetin, curcumin and indole-3-carbinol) could antagonize IS-induced AhR activation and the associated inflammatory response in endothelial cells. The activation of the AhR genomic pathway was assessed by measuring the expression of AhR target genes (*CYP1A1*, *CYP1B1*, and *AHRR*) in endothelial cells and by evaluating AhR-dependent transcriptional activity using a CALUX-AHRE luciferase reporter assay in HG40/6 cells. In parallel, endothelial inflammation was evaluated by analyzing the expression of AhR-related inflammatory genes: *F3*/tissue factor, *PTGS2*/COX-2, *CCL2*/MCP-1, and *CXCL8*/IL-8. Quercetin was the only ligand capable of antagonizing IS-induced AhR transcriptional activity, as well as the upregulation of the endothelial AhR target genes *CYP1A1* and *CYP1B1*. In contrast, galangin, curcumin, and I3C exhibited no inhibitory effects. Moreover, none of the tested dietary AhR ligands suppressed the IS-induced upregulation of endothelial inflammatory genes; instead, they tended to potentiate IS-induced inflammatory responses at high concentrations. In conclusion, among the AhR ligands tested, quercetin was the only one that attenuated IS-induced activation of the AhR genomic pathway in endothelial cells. However, it may also enhance IS-mediated endothelial inflammation, an effect also observed at specific concentrations of galangin, curcumin, and I3C. These findings suggest that the potential beneficial effects of natural dietary AhR ligands should be carefully considered in the context of CKD patients exhibiting high levels of indolic uremic toxins.

## 1. Introduction

Chronic kidney disease (CKD) is a major public health issue affecting approximately three million people in France. CKD is associated with increased mortality, proportional to the decline in renal function [1], mainly due to cardiovascular (CV) causes. This elevated CV risk cannot be explained solely by traditional risk factors (smoking, hypertension, obesity, diabetes, and hypercholesterolemia) [2], and CKD patients often respond poorly to standard therapies targeting these factors. Declining renal function leads to the accumulation of biologically active uremic toxins. Among these, tryptophan-derived uremic toxins, notably indoxyl sulfate (IS) and indole-3-acetic acid (IAA), induce endothelial dysfunction and oxidative stress, impair angiogenesis, activate coagulation, and promote inflammation, thereby increasing CV risk and mortality [3,4,5]. The indolic toxins, IS and IAA, accumulate in CKD and are poorly removed by dialysis [6]. While the physiological concentration of IS is approximately 0.5 µM, uremic concentrations reach 200–250 µM. Circulating indolic toxin levels are predictive of CV events and mortality in CKD patients [7,8]. These toxins exert their biological effects through activation of the aryl hydrocarbon receptor (AhR), as well as a proinflammatory, pro-oxidant, and pro-thrombotic endothelial phenotype [4,7,9].

AhR is a ligand-activated transcription factor involved in xenobiotic detoxification. Its flexible ligand-binding domain allows activation by a wide range of endogenous and exogenous ligands [10,11,12]. In its inactive state, AhR is retained in the cytosol in a complex with HSP90, p23, and XAP2 [13]. Upon ligand binding, AhR translocates to the nucleus, dissociates from its chaperones, and heterodimerizes with ARNT. The AhR/ARNT complex binds AhR response elements (AhRE) and regulates transcription of target genes such as the cytochrome P450 enzymes *CYP1A1* and *CYP1B1*. AhR activation is tightly regulated by nuclear export and proteasomal degradation of the receptor [4,14], as well as by the AhR repressor AHRR, which is upregulated by AhR activation and acts as a transcriptional inhibitor [15].

Beyond its genomic activity, AhR also activates non-genomic inflammatory signaling pathways, leading to activation of NF-κB and AP-1 and promoting a thrombo-inflammatory endothelial phenotype [4,5,7,16]. This pathway induces expression of procoagulant and proinflammatory genes, including *F3* (coding for tissue factor), *PTGS2* (COX2), *CCL2* (MCP-1), and *CXCL8* (IL-8) [4,5,7,16].

CKD patients exhibit elevated circulating AhR agonists, notably indolic toxins, and increased expression of *CYP1A1* and *AHRR* compared with controls [17]. Similarly, increased *CYP1A1* expression is observed in the aortic and myocardial tissues of 5/6 nephrectomized CKD mice and is abolished in AhR-deficient animals [17]. AhR activation correlates with CV events in CKD patients [17]. Consequently, targeting AhR activation by indolic toxins represents a promising strategy to reduce CV complications in CKD patients.

Several synthetic and natural AhR ligands have been described. The pharmacological inhibitor CH223191 blocks AhR nuclear translocation and inhibits both genomic and inflammatory AhR pathways activated by indolic toxins in endothelial cells [16]. No pharmacological inhibitor of AhR is currently available for clinical use. In the present study, we focused on dietary AhR ligands [18,19], which could be rapidly translated into preclinical and clinical settings. Galangin, a flavonoid found in galangal and propolis, exhibits anti-inflammatory and antioxidant properties and antagonizes halogenated hydrocarbon-induced *CYP1A1* transcription in MCF-7 human breast carcinoma cells, although it may act as a partial AhR agonist depending on concentration and cell type [20]. Quercetin, a low-affinity AhR ligand abundant in fruits and vegetables, inhibits NF-κB and MAPK signaling and antagonizes benzo[a]pyrene-induced *CYP1A1* expression in HUVECs [21,22,23]. Curcumin, a lipophilic polyphenol [24], inhibits AhR genomic activation induced by halogenated aromatic hydrocarbons in mouse hepatoma Hepa-1c1c7 cells and suppresses NF-κB and AP-1 signaling pathways [25,26,27]. Indole-3-carbinol (I3C) and its metabolite 3,3′-diindolylmethane (DIM) are cruciferous vegetable-derived AhR ligands. Both compounds have been shown to act as AhR agonists, inducing activation of the AhR genomic pathway in MCF-7 cells [28] and downregulating the expression of proinflammatory proteins [29].

The aim of this study was to evaluate whether dietary AhR ligands can modulate indoxyl sulfate-induced AhR activation and inflammatory responses in endothelial cells.

## 2. Results

### 2.1. Evaluation of Endothelial Cytotoxicity of Dietary AhR Ligands: Galangin, Quercetin, Curcumin, and I3C

**Figure 1:**
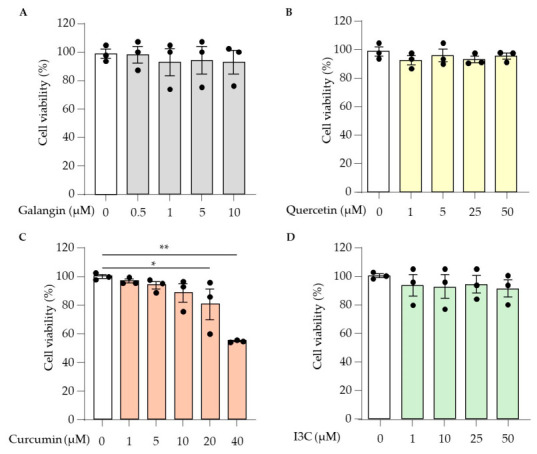
Impact of dietary AhR ligands galangin (**A**), quercetin (**B**), curcumin (**C**), and I3C (**D**) on endothelial cell cytotoxicity evaluated by quantifying LDH release from HUVECs following a 24 h incubation. Data are expressed as mean ± SEM of 3 independent experiments performed on independent cell preparations. Values were compared using the Friedman test followed by Dunn’s post hoc test. * *p* ≤ 0.05, ** *p* ≤ 0.01.

Galangin, quercetin, and I3C did not exhibit endothelial cytotoxicity at any tested concentrations (0.5–10 µM, 1–50 µM, and 1–50 µM, respectively) after 24 h of stimulation (Figure 1A,B,D). Curcumin did not induce endothelial cytotoxicity at concentrations of 1, 5, or 10 µM; however, a cytotoxic effect exceeding 10% was observed from 20 µM onward (Figure 1C). Consequently, non-cytotoxic concentrations of AhR ligands were selected for subsequent experiments.

### 2.2. In Vitro Effects of Dietary AhR Ligands on Activation of the AhR Genomic Pathway in Endothelial Cells

**Figure 2:**
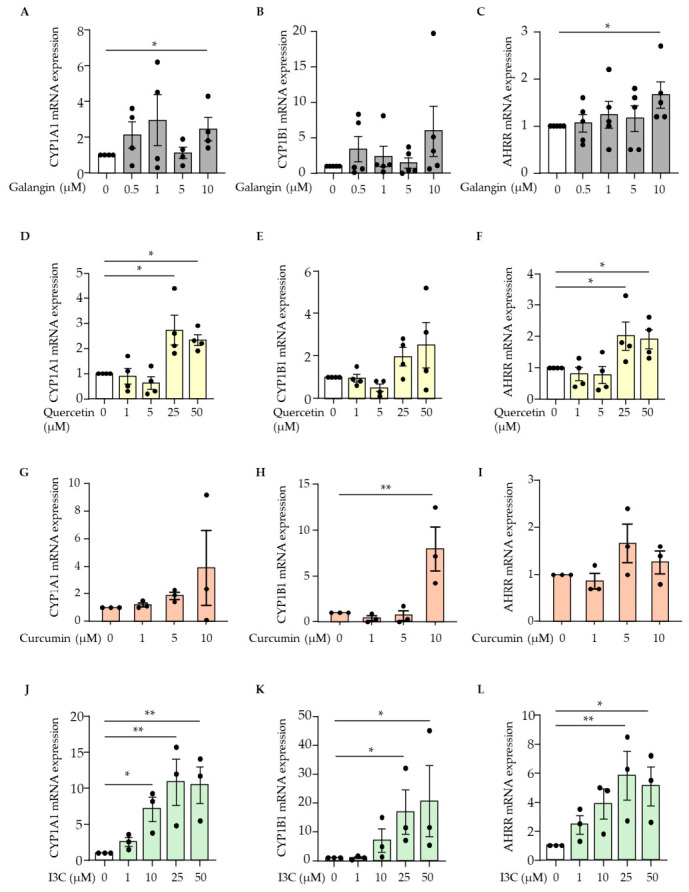
Dose–response effect of dietary AhR ligands on the expression of AhR target genes *CYP1A1* (**A**,**D**,**G**,**J**), *CYP1B1* (**B**,**E**,**H**,**K**), and *AHRR* (**C**,**F**,**I**,**L**) measured by RT-qPCR in endothelial cells incubated for 24 h with galangin (**A**–**C**), quercetin (**D**–**F**), curcumin (**G**–**I**), or I3C (**J**–**L**) at different concentrations. Data are expressed as mean ± SEM of 3–4 independent experiments performed on independent cell preparations. Values were compared by ANOVA followed by Fisher’s LSD test. * *p* ≤ 0.05, ** *p* ≤ 0.01.

We investigated the effects of dietary AhR ligands on the AhR genomic pathway in endothelial cells by measuring the expression of AhR target genes *CYP1A1*, *CYP1B1*, and *AHRR* in the absence of indolic toxin. Galangin increased the expression of the AhR target genes *CYP1A1* and *AHRR* at 10 µM but had no effect at lower concentrations (Figure 2A–C). Quercetin had no effect on the AhR target genes at low concentrations (1 and 5 µM) but significantly increased the expression of *CYP1A1* and *AHRR* at high concentrations (25 and 50 µM) (Figure 2D–F). Curcumin increased the expression of *CYP1B1* only at 10 µM (Figure 2H) and did not significantly modify the expression of *CYP1A1* or *AHRR* (Figure 2G–I). In contrast, I3C at high concentrations (25 and 50 µM) induced an increased expression of the three AhR target genes (Figure 2J–L).

### 2.3. Effect of Dietary AhR Ligands on Indoxyl Sulfate-Induced AhR Target Gene Expression in Endothelial Cells

**Figure 3:**
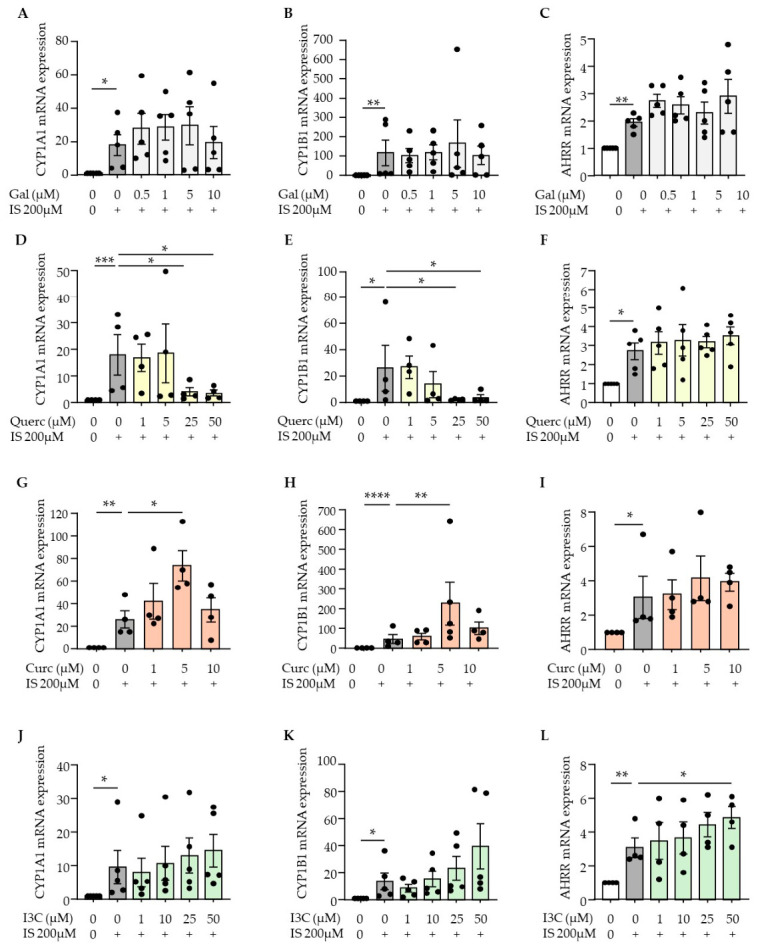
Dose–response effect of dietary AhR ligands on endothelial AhR target gene upregulation induced by indoxyl sulfate**.** The expression of AhR target genes *CYP1A1* (**A**,**D**,**G**,**J**), *CYP1B1* (**B**,**E**,**H**,**K**), and *AHRR* (**C**,**F**,**I**,**L**) was measured by RT-qPCR following a 24 h stimulation of endothelial cells with 200 µM indoxyl sulfate (IS) in the presence of galangin (Gal) (**A**–**C**), quercetin (Querc) (**D**–**F**), curcumin (Curc) (**G**–**I**), and indole-3 carbinol (I3C) (**J**–**L**) at different concentrations. Data are expressed as mean ± SEM of 4–5 independent experiments performed on independent cell preparations. Values were compared by ANOVA followed by Fisher’s LSD test. * *p* ≤ 0.05, ** *p* ≤ 0.01, *** *p* ≤ 0.001, **** *p* ≤ 0.0001.

We next examined the effects of dietary AhR ligands on the activation of AhR target genes induced by the indolic toxin indoxyl sulfate (IS). As expected, IS increased the expression of *CYP1A1*, *CYP1B1*, and *AHRR*, indicating activation of the AhR genomic pathway (Figure 3). Galangin, curcumin, and I3C did not inhibit IS-induced activation of the AhR target genes (Figure 3). Rather, curcumin amplified the IS-induced expression of *CYP1A1* and *CYP1B1* at 5 µM (Figure 3G–I), and I3C amplified *AHRR* expression at 50 µM (Figure 3L). In contrast, quercetin was the only compound to inhibit IS-induced activation of the genomic AhR pathway, decreasing *CYP1A1* and *CYP1B1* upregulation at 25 and 50 µM (Figure 3D,E) without affecting *AHRR* expression (Figure 3F).

### 2.4. Effect of Dietary AhR Ligands on AhR-Dependent Transcriptional Activity

**Figure 4:**
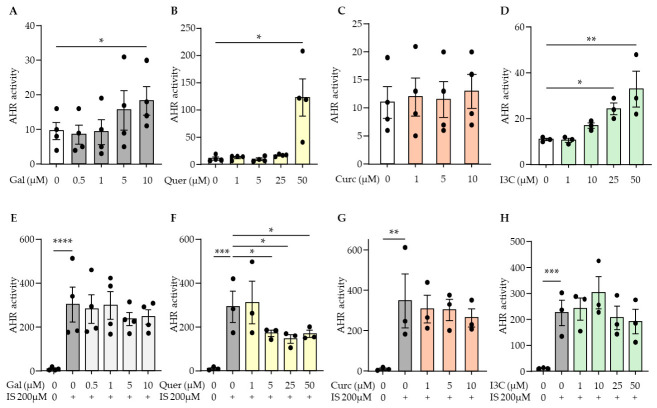
Dose–response effect of dietary AhR ligands on AhR transcriptional activity in the absence and presence of indoxyl sulfate. AhR-dependent transcriptional activity was assessed using a CALUX (Chemically Activated LUciferase gene eXpression) reporter assay following 24 h exposure to the dietary AhR ligands galangin (Gal) (**A**,**E**), quercetin (Quer) (**B**,**F**), curcumin (Curc) (**C**,**G**), and indole-3-carbinol (I3C) (**D**,**H**) at different concentrations, without (**A**–**D**) or with (**E**–**H**) 200 µM indoxyl sulfate (IS). Data are expressed as mean ± SEM of 3–4 independent experiments performed on independent cell preparations. Values were compared by ANOVA followed by Fisher’s LSD test. * *p* ≤ 0.05, ** *p* ≤ 0.01, *** *p* ≤ 0.001, **** *p* ≤ 0.0001.

To further assess the effect of AhR ligands on the AhR genomic pathway, we studied the AhR-dependent transcriptional activity using a CALUX reporter assay, based on AhRE-driven luciferase expression in HG40/6 cells (Figure 4). In the absence of indoxyl sulfate, the effects of AhR ligands were both dose- and ligand-dependent (Figure 4A–D). Galangin (Figure 4A), quercetin (Figure 4B), and I3C (Figure 4D) increased AhR transcriptional activity only at the highest concentrations tested (10, 50, and 25 and 50 µM, respectively), with no significant effect at lower concentrations. In contrast, curcumin did not significantly affect AhR transcriptional activity at any of the concentrations tested (Figure 4C).

We next examined the effects of dietary AhR ligands on the activation of AhR transcriptional activity induced by indoxyl sulfate (Figure 4E–H). As expected, IS markedly increased AhR transcriptional activity, which was inhibited only by quercetin at 5, 25 and 50 µM (Figure 4F), whereas the other dietary AhR ligands galangin, curcumin, and I3C had no effect (Figure 4E,G,H).

### 2.5. In Vitro Effects of Dietary AhR Ligands on IS-Induced Inflammatory Gene Expression in Endothelial Cells

**Figure 5:**
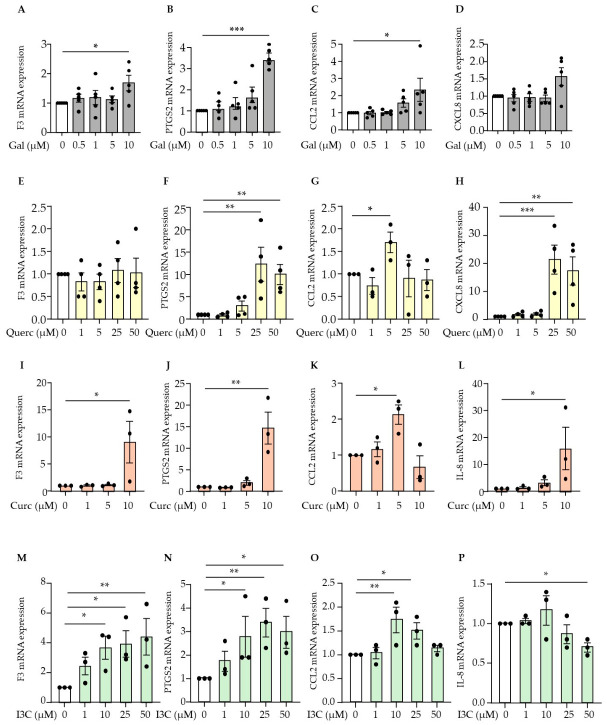
Dose–response effect of dietary AhR ligands on endothelial inflammatory gene expression. The expression of inflammatory genes *F3* (**A**,**E**,**I**,**M**), *PTGS2* (**B**,**F**,**J**,**N**), *CCL2* (**C**,**G**,**K**,**O**) and *CXCL8* (**D**,**H**,**L**,**P**) was measured by RT-qPCR in endothelial cells incubated for 24 h with galangin (Gal) (**A**–**D**), quercetin (Querc) (**E**–**H**), curcumin (Curc) (**I**–**L**), and indole-3-carbinol (I3C) at different concentrations. Data are expressed as mean ± SEM of 3–4 independent experiments performed on independent cell preparations. Values were compared by ANOVA followed by Fisher’s LSD test. * *p* ≤ 0.05, ** *p* ≤ 0.01, *** *p* ≤ 0.001.

We finally evaluated the effects of dietary AhR ligands on endothelial inflammatory genes related to AhR activation by measuring the expression of *F3* (encoding tissue factor), *PTGS2* (COX-2), *CCL2* (MCP-1), and *CXCL8* (IL-8). In the absence of indolic toxin, galangin exerted an agonistic effect on inflammatory genes only at 10 µM (Figure 5A–D). Quercetin strongly increased the expression of *PTGS2* and *CXCL8* at 25 and 50 µM (Figure 5F,H) and of *CCL2* at 5 µM (Figure 5G) but did not affect *F3* expression (Figure 5E). Curcumin markedly increased the expression of *F3, PTGS2*, and *CXCL8* at 10 µM (Figure 5I,J,L), and of *CCL2* at 5 µM (Figure 5K). I3C upregulated the expression of *F3, PTGS2*, and *CCL2* from 10 µM (Figure 5M–O), but not *CXCL8*, whose expression was even inhibited at 50 µM (Figure 5P).

**Figure 6:**
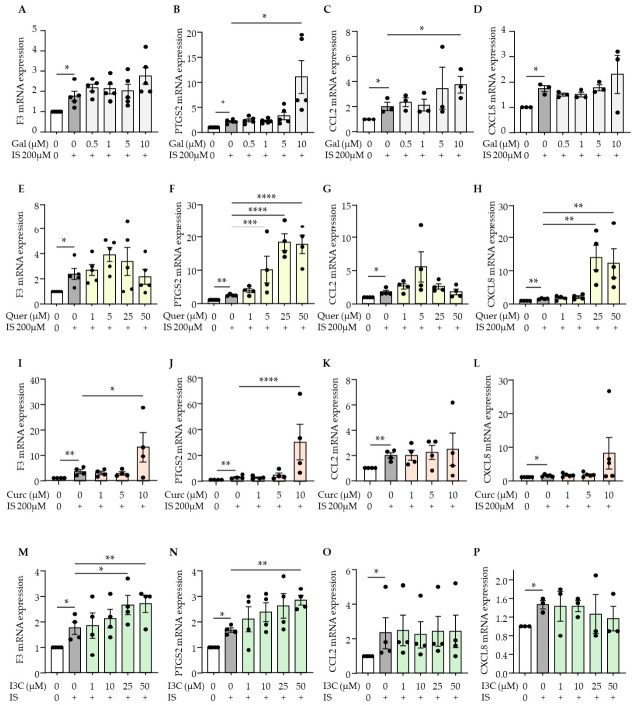
Dose–response effect of dietary AhR ligands on endothelial inflammatory gene upregulation induced by indoxyl sulfate. The expression of inflammatory genes *F3*/TF (**A**,**E**,**I**,**M**), *PTGS2/*COX2 (**B**,**F**,**J**,**N**), *CCL2/*MCP-1 (**C**,**G**,**K**,**O**), and *CXCL8/*IL-8 (**D**,**H**,**L**,**P**) was measured by RT-qPCR following a 24 h stimulation of endothelial cells with 200 µM indoxyl sulfate (IS) in the presence of galangin (Gal) (**A**–**D**), quercetin (Quer) (**E**–**H**), curcumin (Curc) (**I**–**L**), and indole-3-carbinol (I3C) (**M**–**P**) at different concentrations. Data are expressed as mean ± SEM of 3–5 independent experiments performed on independent cell preparations. Values were compared by ANOVA followed by Fisher’s LSD test. * *p* ≤ 0.05, ** *p* ≤ 0.01, *** *p* ≤ 0.001, **** *p* ≤ 0.0001.

In the presence of IS, the endothelial expression of AhR-related inflammatory genes was upregulated (Figure 6) and was not reduced by any of the dietary AhR ligands (Figure 6). Instead, galangin at 10 µM amplified the IS-induced expression of *PTGS2* and *CCL2* (Figure 6B,C), quercetin at 25 and 50 µM amplified *PTGS2* and *CXCL8* expression (Figure 6F,H), and curcumin at 10 µM (Figure 6I,J) and I3C at 50 µM (Figure 6M,N) amplified the IS-induced expression of *F3* and *PTGS2*.

**Figure 7:**
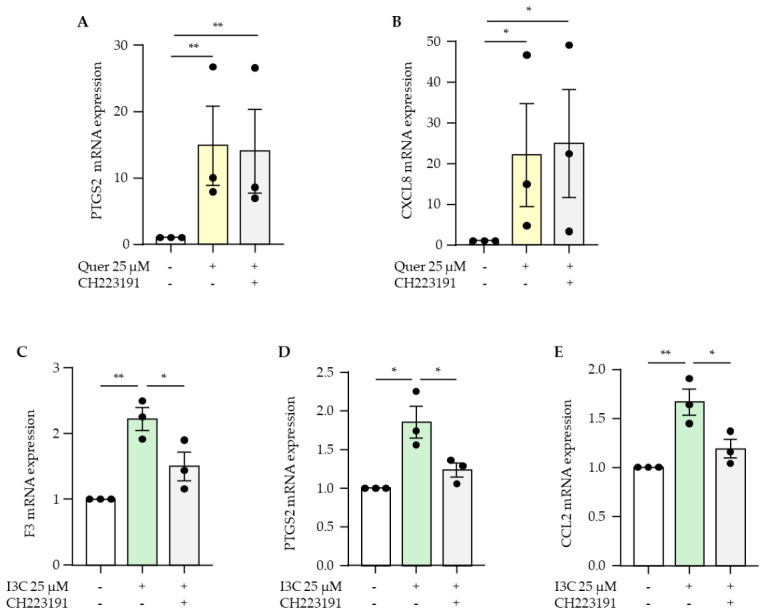
Effect of the pharmacological AhR inhibitor CH223191 on the endothelial inflammatory phenotype induced by the dietary AhR ligands quercetin and indole-3-carbinol. The expression of inflammatory genes *PTGS2* (**A**,**D**), *CXCL8* (**B**), *F3* (**C**), and *CCL2* (**E**) was measured by RT-qPCR in endothelial cells incubated for 24 h with 25 µM quercetin (Quer) (**A**,**B**) and 25 µM indole-3-carbinol (I3C) (**C**–**E**) in the presence of the AhR inhibitor CH223191 at 0.5 µM. Data are expressed as mean ± SEM of 3 independent experiments performed on independent cell preparations. Values were compared by ANOVA followed by Fisher’s LSD test. * *p* ≤ 0.05, ** *p* ≤ 0.01.

To investigate whether the inflammatory phenotype induced by dietary AhR ligands involves AhR-dependent or AhR-independent mechanisms, we examined the effect of the pharmacological AhR inhibitor CH223191 on the upregulation of inflammatory genes induced by high concentrations (25 µM) of quercetin and I3C. The effects of CH223191 were ligand-dependent, with quercetin and I3C exhibiting distinct response patterns. CH223191 did not affect the quercetin-induced upregulation of *PTGS2* and *CXCL8*, either in the absence (Figure 7A,B) or presence of IS (Supplementary Figure S1A,B). In contrast, CH223191 significantly inhibited the I3C-induced upregulation of *F3*, *PTGS2*, and *CCL2*, both in the absence (Figure 7C–E) and presence of IS (Supplementary Figure S1C–E).

## 3. Discussion

The aim of this study was to identify in vitro dietary AhR ligands able to inhibit endothelial AhR activation induced by uremic indolic toxins such as indoxyl sulfate (IS). AhR functions as a receptor for numerous ligands, including indolic toxins, as well as a transcription factor and signaling molecule. As a transcription factor, AhR translocates to the nucleus, dimerizes with ARNT, and binds to the promoter regions of its target genes, such as *CYP1A1* and *CYP1B1,* thereby inducing their expression through the so-called genomic pathway. The AhR/ARNT complex also upregulates the transcription of its repressor, AHRR, which acts as a transcriptional inhibitor in a negative feedback loop regulating AhR [15].

In this context, AhR activation has been reported to be differentially modulated by dietary compounds depending on the nature of the ligand and the cellular model. Galangin, quercetin and curcumin have been shown to inhibit the genomic AhR pathway activated by halogenated hydrocarbons (TCDD) or benzo[α]pyrene (BaP) in cancer cells or animal models [20,23,25]. I3C is described as a weak ligand of AhR that promotes enhanced detoxification through activation of the genomic pathway in intestinal mucosae [19]. Here, we found that four dietary AhR ligands—galangin, curcumin, quercetin, and I3C—induced distinct, concentration-dependent expression profiles of AhR target gene upregulation in endothelial cells in the absence of uremic toxins. High concentrations of I3C and, to a lesser extent, quercetin and galangin upregulated AhR target genes, suggesting they promote a canonical activation of the AhR pathway. Consistent with these findings, galangin, quercetin, and I3C increased AhR-dependent transcriptional activity in the CALUX reporter assay, which measures AhR/ARNT binding to AhR response elements (AhREs).

In contrast, curcumin modulated only one of the endothelial AhR target genes, indicating selective or non-canonical modulation of AhR signaling, which was confirmed by an absence of effect on AhR transcriptional activity.

AhR antagonism is also ligand-specific. For example, in HepG2 liver cells, jasmones weakly activate the genomic AhR pathway and antagonize AhR activation by TCDD and BaP but not by FICZ [30]. Specifically, they do not prevent AhR nuclear translocation but inhibit AhR-ARNT interaction (necessary for regulating target genes of the genomic pathway) when activated by TCDD. Thus, an agonist can act antagonistically in the presence of another AhR ligand in a ligand-dependent manner. We therefore tested whether the dietary AhR ligands can antagonize the effect of IS and observed different profiles. Galangin did not alter the IS-mediated increase in AhR target genes or AhR transcriptional activity, indicating that it does not antagonize the canonical AhR activation induced by IS. Neither curcumin nor I3C inhibited the activation of AhR transcriptional activity mediated by IS; rather, they dose-dependently amplified the upregulation of some endothelial AhR target genes. In contrast, quercetin at high concentrations, despite its activating effect on the genomic AhR pathway when used alone, was the only ligand able to antagonize the IS-induced AhR transcriptional activity as well as the upregulation of endothelial AhR target genes *CYP1A1* and *CYP1B1*. However, these concentrations are higher than those achievable through oral supplementation (3.5–5 µM) [19]. These data suggest that quercetin exerts a dose-dependent dual activity on the AhR genomic pathway, acting as an agonist in the absence of indoxyl sulfate while selectively antagonizing IS-induced AhR activation. This highlights that an AhR ligand can display dose-dependent agonistic activity as well as antagonistic properties in the presence of another AhR ligand, such as indoxyl sulfate. However, the translation of these results to the clinical setting should be approached with caution, as the concentrations required for quercetin to exert inhibitory effects on AhR activation may be difficult to achieve in vivo owing to its limited oral bioavailability and extensive metabolism, despite its slow rate of elimination [19,31]. A more comprehensive evaluation of the biological activities of its major metabolites, including glucuronides, sulfates, and methylated derivatives such as isorhamnetin, would be of particular interest.

AhR can also function as a signaling molecule, activating inflammatory pathways such as NF-κB and AP-1, which lead to the overexpression of thrombo-inflammatory genes like tissue factor/*F3*, COX-2/*PTGS2,* MCP-1/*CCL2* or IL-8/*CXCL8* [7,16,32]. We have shown that the IS-induced upregulation of *F3* and *PTGS2* in endothelial cells is mediated by p38 MAPK/NF-κB activation downstream of AhR [7,16], whereas the upregulation of *CCL2* and *CXCL8* is mediated via TAK1/p38 MAPK/JUN signaling [32]. This AhR-driven inflammatory phenotype induced by indolic toxins may have clinical consequences, such as cardiovascular or hemodialysis arteriovenous access complications in CKD patients [7,32]. We therefore investigated whether dietary AhR ligands could reduce IS/AhR-mediated upregulation of endothelial inflammatory genes. However, none of the tested ligands was able to inhibit the IS-induced AhR-related inflammatory pathway. Rather, at certain concentrations, these AhR ligands upregulated endothelial inflammatory genes and amplified the effect of IS on their expression. To gain insight into the mechanisms underlying the proinflammatory effects of dietary AhR ligands, we selected quercetin and I3C as representative compounds, as both induce inflammatory responses at high concentrations while exhibiting distinct effects on the genomic AhR pathway. We then examined the effect of pharmacological AhR inhibition on inflammatory responses. The use of the pharmacological AhR inhibitor CH223191 revealed distinct mechanisms. Whereas CH223191 failed to prevent the inflammatory response induced by quercetin, irrespective of the presence of IS, it markedly inhibited the upregulation of inflammatory genes in response to I3C. These findings indicate that dietary AhR ligands activate distinct signaling pathways leading to inflammatory gene expression: the inflammatory response elicited by I3C is largely AhR-dependent, whereas quercetin appears to act through an AhR-independent mechanism and might, at high concentrations, activate proinflammatory transcription factors, thereby contributing to the observed inflammatory response. These findings highlight the complexity of the biological actions of dietary AhR ligands and emphasize that the inflammatory responses they induce are ligand- and concentration-specific. The proinflammatory effects observed with dietary AhR ligands at specific concentrations in the present study may appear paradoxical, as these compounds have been reported to exert anti-inflammatory effects in other cell types [24,25,26,27,28]. However, the biological effects of AhR ligands are known to depend on multiple factors, including ligand structure, concentration, and cell type. In particular, some dietary AhR ligands, such as galangin and I3C, have been reported to act as partial AhR agonists in a concentration-dependent manner [20,28], consistent with the effects observed in our study. Our data obtained using pharmacological AhR inhibition further suggest that the inflammatory responses induced by dietary AhR ligands do not rely on a common mechanism but may occur through either AhR-dependent or AhR-independent pathways. Regarding quercetin, our results differ from previous studies performed in cultured endothelial cells, in which quercetin was reported to reduce the expression of inflammatory genes [21,22]. However, these studies used quercetin at concentrations different from those tested in the present study, and the inflammatory genes investigated also differed from those analyzed here. Context-dependent effects of quercetin have also been shown in humans. Quercetin can exert sex-specific proinflammatory actions, as reported in female patients with coronary artery disease, where it paradoxically increased inflammatory markers [33]. Together, these findings indicate that the pro- or anti-inflammatory effects of dietary AhR ligands are concentration- and context-dependent and may also vary according to the ligand itself and the presence of other AhR agonists.

Our study has several limitations. First, it was performed exclusively in vitro, which does not allow definitive conclusions regarding the effects of AhR ligands in patients with CKD. In addition, the oral bioavailability of these compounds is limited [19], their protein binding may be high [34], and the peak plasma concentrations achievable in humans are lower than those used in vitro [19,35]. Although ligand concentrations were selected to avoid cytotoxic effects in endothelial cells in vitro and to ensure that the lowest concentrations tested could be reached in vivo, the highest concentrations (>10µM) of dietary ligands are unlikely to be achieved in human plasma [19,34,35]. Moreover, the metabolic fate of these compounds in CKD patients remains insufficiently understood. Our conclusions are also based exclusively on gene expression analyses; therefore, additional studies assessing protein expression and functional outcomes are needed to further validate these findings. Finally, our in vitro study does not account for in vivo concentrations, ligand metabolism, or the biological activities of their metabolites, further supporting the need for in vivo studies to evaluate the effects of dietary AhR ligands under physiologically relevant conditions.

Some dietary AhR ligands have previously been tested in CKD patients. In particular, curcumin and quercetin have been investigated for their potential nephroprotective effects in advanced CKD, notably in relation to their ability to reduce uremic toxin levels [36,37]. Overall, our findings, despite their limitations, as well as the complexity of CKD patient physiology, highlight the need for a more thorough evaluation of the potential benefits of dietary AhR ligands in the context of CKD.

## 4. Conclusions

Among dietary AhR ligands, quercetin was the only compound to antagonize IS-induced activation of the AhR genomic pathway, while also promoting endothelial inflammation. In contrast, curcumin, galangin, and I3C did not counteract IS-induced activation of AhR genomic or inflammatory pathways and even appeared to exacerbate its effects at certain concentrations. Overall, these findings highlight the complex responses to dietary AhR ligands in the context of CKD and underscore the need for further studies when considering the use of dietary AhR ligands in CKD patients with elevated levels of indolic uremic toxins.

## 5. Materials and Methods

### 5.1. Culture and Stimulation of Endothelial Cells

Human umbilical vein endothelial cells (HUVECs) were obtained from Lonza (France) and then cultured in EGM-2 medium (Lonza, Colmar, France) containing 2% fetal bovine serum under standard cell culture conditions (humidified atmosphere, 37 °C, 5% CO_2_). The cells were incubated for 24 h with dietary AhR ligands—galangin, quercetin, curcumin, and I3C—with and without 200 µM IS (all from Merck-Sigma-Aldrich Chimie, Saint-Quentin Fallavier, France). Each compound was compared to its control at the same dilution: DMSO for galangin and quercetin, and ethanol for curcumin and I3C. Since IS is commercially available as a potassium salt, we used potassium chloride (KCl) as a control at the same dilution as IS (1/1000). A concentration of 200 µM IS corresponds to the IS levels to which patients with severe CKD are exposed. To inhibit AhR activation, some experiments were performed in the presence of the pharmacological AhR inhibitor CH223191 (Merck-Sigma-Aldrich Chimie, Saint-Quentin Fallavier, France) at 0.5 µM.

### 5.2. Cytotoxicity Studies

The cytotoxicity of the dietary ligands on HUVECs was assessed using the CyQUANT LDH Cytotoxicity Assay Kit (ThermoFisher, Illkirch, France) after 24 h of stimulation.

### 5.3. RNA Extraction and RT-qPCR Experiments

After 24 h of stimulation, endothelial cells were lysed using RLT buffer (Qiagen, Courtaboeuf, France) supplemented with β-mercaptoethanol. Total RNA was extracted using the RNeasy Mini Kit (Qiagen, Courtaboeuf, France). Reverse transcription (RT) was performed on 500 ng of total RNA using the Takara PrimeScript RT Reagent Kit (Takara, Saint-Germain-en-Laye, France). Quantitative polymerase chain reaction (qPCR) was performed on 10 ng of cDNA using the TB Green Premium kit (Takara, Saint-Germain-en-Laye, France). We quantified the following target genes: *PTGS2* (encoding COX-2), *F3* (encoding tissue factor), *CXCL8* (encoding IL-8), *CCL2* (encoding MCP-1), *CYP1A1*, *CYP1B1*, and *AHRR*. The housekeeping gene *HPRT* was used for normalization of target gene expression values. Primer sequences are available in the Supplementary Materials (Supplemental Table S1).

### 5.4. Assessment of AhR Transcriptional Activity by CALUX (Chemically Activated Luciferase Gene Expression) Reporter Assay

The transcriptional activity of AhR was assessed using a CALUX reporter assay. HG40/6 cells (a gift from Dr. Gary H. Perdew, Pennsylvania State University, Pennsylvania, USA), derived from the HepG2 human hepatoma cell line, stably transfected with a reporter construct containing AhR response elements (AhREs) upstream of the luciferase gene were used, as previously described [38]. Cells were exposed for 24 H to dietary AhR ligands, in the presence or absence of 200 µM IS, or to the potent AhR agonist FICZ at 10 nM. Luminescence, reflecting luciferase activity (measured in relative light units, RLUs), and viable cell number, determined by a fluorescence-based method, were assessed using the ONE-Glo™ + Tox Luciferase & Cell Viability assay (Promega, Charbonnières-les-Bains, France) and quantified with a Glomax^®^ plate reader (Promega, Charbonnières-les-Bains, France). Luciferase activity was normalized to the relative number of viable cells. AhR transcriptional activity was expressed as a percentage of the response induced by 10 nM FICZ (% of FICZ 10 nM), calculated by dividing the normalized RLU of each sample by the normalized RLU obtained with FICZ and multiplying by 100.

### 5.5. Statistical Analyses

Statistical analyses were performed with Prism software (v10, GraphPad, CA, San Diego, CA, USA). Statistical significance was assessed using ANOVA or the Friedman test, followed by appropriate post hoc tests (Fisher’s or Dunn’s), depending on data distribution and pairing. Data are expressed as mean ± SEM of independent experiments performed on independent cell preparations. A *p*-value ≤ 0.05 was considered significant.

## Data Availability

The original contributions presented in this study are included in the article/Supplementary Material. Further inquiries can be directed to the corresponding author.

## Supplementary Materials

The following supporting information can be downloaded at https://www.mdpi.com/article/10.3390/toxins18070298/s1. Table S1: Sequences of primers (Invitrogen, Life Technologies, Saint-Aubin, France) used in RT-qPCR experiments. Figure S1: Effect of the pharmacological AhR inhibitor CH223191 on the endothelial inflammatory phenotype induced by quercetin and indole-3-carbinol in the presence of indoxyl sulfate.
