# Supplementary material for: Effect of Dietary Aryl Hydrocarbon Receptor Ligands on Indoxyl Sulfate-Induced Endothelial Activation
Source: Toxins (Basel). 2026 Jul 10;18(7):298. doi: 10.3390/toxins18070298 (PMC13417053; doi:10.3390/toxins18070298)
Supplement: Supplementary file 1 [file toxins-18-00298-s001.zip › toxins-4319341-supplementary.pdf]

Supplementary Materials: Effect of Dietary Aryl Hydrocarbon Receptor Ligands on Indoxyl Sulfate-Induced Endothelial Activation

Flora Lefevre, Rania Chermiti, Julien Cebile, Nathalie McKay, Stanislas Bataille, Stéphane Burtey and Laetitia Dou

Table S1. Sequences of primers (Invitrogen, Life Technologies, Saint-Aubin, France) used in RT-qPCR experiments.

Gene	Primer forward	Primer reverse
CCL2	5′TCTGTGCCTGCTGCTCATAG3′	5′CAGATCTCCTTGGCCACAAT3′
CXCL8	5′CTCCACAACCCTCTGCAC3′	5′TGCCAAGGAGTGCTAAAG3′
F3	5′-GCCCTCCCTTTCCTGCCATAGA-3′	5′-CCTCCCGGTAGGAAACTCCG-3′
PTGS2	5′ ATTGACCAGAGCAGGCAGAT 3′	5′ ATTGACCAGAGCAGGCAGAT 3′
CYP1A1	5′GACAGATCCCATCTGCCCTA3′	5′ATAGCACCATCAGGGGTGAG3′
CYP1B1	5′TGATGGACGCCTTTATCCTC3′	5′CCACGACCTGATCCAATTCT3′
AHRR	5′GAAGGAGCAGCAGAGAGAGC3′	5′CTTTGTGGGTCCTGGAGTCT3′
HPRT	5′GGATTATACTGCCTGACCAAGGAAAGC3′	5′GAGCTATTGTAATGACCAGTCAACAGG3′

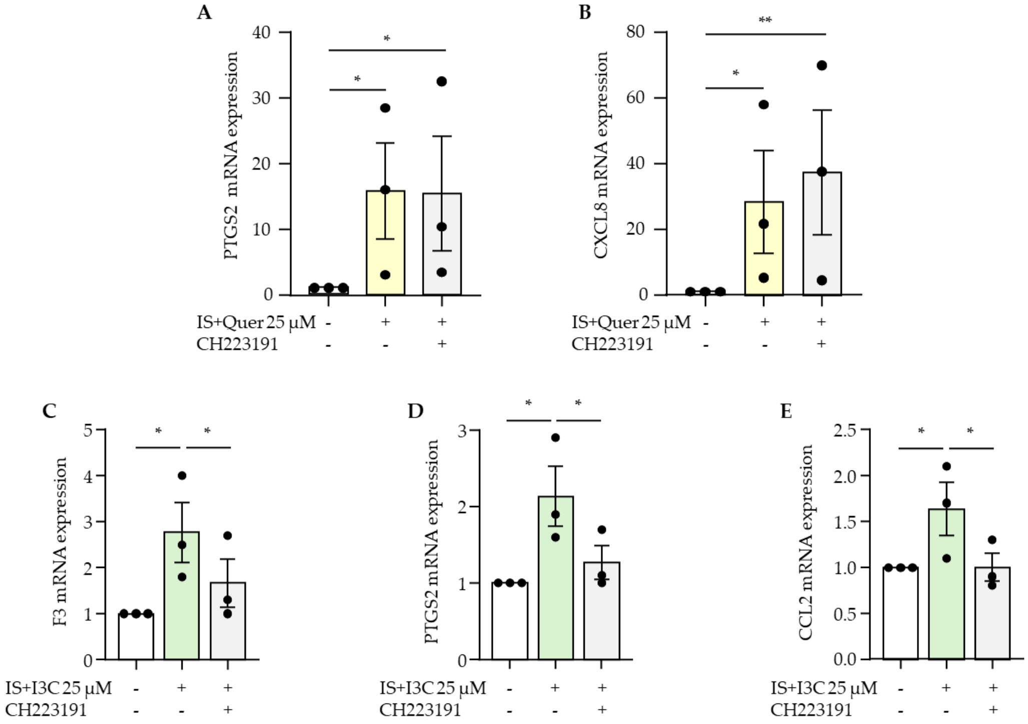

Figure S1. Effect of the pharmacological AhR inhibitor CH223191 on the endothelial inflammatory phenotype induced by quercetin and indole-3-carbinol in the presence of indoxyl sulfate. The expression of inflammatory genes *PTGS2* (A, D), *CXCL8* (B), F3 (C), and *CCL2* (E) was measured by RT-qPCR in endothelial cells incubated during 24 hours with 25 µM quercetin (Quer) (A, B) and 25 µM indole-3-carbinol (I3C) (C, D, E) in presence of the AhR inhibitor CH223191 at 0.5 µM. Data are expressed as mean ± SEM of 3 independent experiments performed on independent cell preparations. Values were compared by ANOVA followed by Fisher's LSD test. *$p \leq 0.05$, **$p \leq 0.01$.
